# Impact of Comorbidity, Race, and Marital Status in Men Referred for Prostate Biopsy with PSA >20 ng/mL: A Pilot Study in High-Risk Patients

**DOI:** 10.1155/2014/362814

**Published:** 2014-08-24

**Authors:** Zachary Klaassen, Roberto Muller, Qiang Li, Alexander J. Tatem, Sherita A. King, Stephen J. Freedland, Rabii Madi, Martha K. Terris, Kelvin A. Moses

**Affiliations:** ^1^Department of Surgery, Section of Urology, Charlie Norwood Veteran's Administration Medical Center, Augusta, GA 30904, USA; ^2^Department of Surgery, Section of Urology, Medical College of Georgia, Georgia Regents University, Augusta, GA 30912, USA; ^3^Section of Surgery, Division of Urology, Durham Veteran's Administration Medical Center, Durham, NC 27705, USA; ^4^Departments of Surgery and Pathology, Division of Urology, Duke University School of Medicine, Durham, NC 27710, USA; ^5^Department of Urologic Surgery, Vanderbilt University, A-1302 Medical Center North, Nashville, TN 37232, USA

## Abstract

*Objective*. To assess the impact of comorbidity, race, and marital status on overall survival (OS) among men presenting for prostate biopsy with PSA >20 ng/mL. *Methods*. Data were reviewed from 2000 to 2012 and 78 patients were included in the cohort. We analyzed predictors of OS using a Cox proportional hazards model and the association between Charlson Comorbidity Index (CCI) score and PCa diagnosis or high-grade cancer using logistic regression and multinomial regression models, respectively. *Results*. The median age of patients was 62.5 (IQR 57–73) years. Median CCI was 3 (IQR 2–4), 69% of patients were African American men, 56% of patients were married, and 85% of patients had a positive biopsy. CCI (HR 1.52, 95% CI 1.19, 1.94), PSA (HR 1.62, 95% CI 1.09, 2.42), and Gleason sum (HR 2.04, 95% CI 1.17, 3.56) were associated with OS. CCI was associated with Gleason sum 7 (OR 4.06, 95% CI 1.04, 15.89) and Gleason sum 8–10 (OR 4.52, 95% CI 1.16, 17.54) PCa. *Conclusions*. CCI is an independent predictor of high-grade disease and worse OS among men with PCa. Race and marital status were not significantly associated with survival in this cohort. Patient comorbidity is an important component of determining the optimal approach to management of prostate cancer.

## 1. Introduction

In 2013 there will be an estimated 238,590 new cases of prostate cancer (PCa) in the United States resulting in an estimated 29,720 deaths [1]. Approximately 15–30% of new PCa diagnoses are classified as high risk [2], defined by D'Amico as PSA >20 ng/mL, biopsy Gleason score 8–10 PCa, or clinical stage ≥T2c disease [3]. There is controversy regarding the optimal treatment for men in the various risk classifications; however recently updated AUA guidelines support screening for PCa in men who have at least a 10-year life expectancy [4]. One tool that allows identifying patients with significant comorbidities is the Charlson Comorbidity Index (CCI), which computes a weighted score based on 17 comorbidity groups to estimate the relative risk of 10-year mortality [5].

In addition to the physiologic status of the patient, social constructs such as race and marital status play an important role in the diagnosis and treatment of PCa. It has previously been established that African American (AA) men present with more advanced disease, are administered less aggressive treatment regimens, have shorter progression-free survival following treatment, and have more treatment-related side effects and diminished quality of life compared to the general population [6–12]. Furthermore, there is evidence that AA men with PCa are less likely to be married and are more likely to be uninsured [13, 14]. Recent studies have suggested that marital status may also have an impact on cancer outcomes. Specifically, some studies suggest that married men have better survival outcomes in patients undergoing radical cystectomy for bladder cancer [15–17] or radical prostatectomy (RP) for PCa [18] compared to patients who are single, divorced, or widowed (SDW).

Results indicating that outcomes vary based upon comorbidity, race, and marital status are largely based on surgical databases [18]. Ideally, studies should analyze risk factors of men at increased risk of worse PCa outcomes before diagnosis and assignment to a particular treatment. We investigated the impact of comorbidities, race, and marital status on the risk of advanced PCa and overall survival (OS) among high-risk men based on high PSA levels referred for prostate biopsy, with emphasis on OS. We hypothesized that men with extensive comorbidities, AA men, and SDW men would have higher Gleason score and poorer OS compared to healthier, Caucasian, and married men, respectively.

## 2. Materials and Methods

### 2.1. Study Population

After receiving institutional review board approval, we performed a retrospective evaluation of a prospective database of patients referred to the CNVAMC for prostate biopsy in Augusta, GA; the CNVAMC is an equal access, primary care based system. Between January 1, 2000, and August 31, 2012, 1979 patients underwent prostate biopsy for cause (abnormal digital rectal examination and/or increased PSA values). Within this group, 78 patients (3.9%) had a PSA >20 ng/mL at the time of biopsy and formed the study cohort. The variables of interest included age, CCI score, race (AA versus Caucasian), marital status (married versus SDW), history of previous biopsy, history of prior PSA, clinical tumor (T) classification, body mass index (BMI), biopsy Gleason score, and treatment received. Treatment modality was according to the discretion of the patient and treating physician. Biochemical recurrence (BCR) was defined as >0.2 ng/mL for patients after RP and as a rise by 2 ng/mL or more above the nadir PSA for patients undergoing external beam radiotherapy (EBRT) according to American Society for Therapeutic Radiology and Oncology (ASTRO) Phoenix Consensus [19]. Cause of death was extrapolated from chart review by a genitourinary oncologist; the advantage of the VA online medical record is that cause of death is recorded or obtained (if death occurred outside of the VA system).

### 2.2. Statistical Analysis

Population demographics of the overall cohort were described using median and interquartile ranges and tabulations of relative frequency of data. OS was estimated using the Kaplan-Meier method, and we analyzed predictors of OS among the 66 men diagnosed with PCa using a multivariable Cox proportional hazards model, which included the variables age, race, marital status, PSA (log-transformed), Gleason score, and CCI. We tested the association between CCI score and PCa diagnosis or high-grade cancer using logistic regression and multinomial regression models, respectively. The outcomes for this model were Gleason score (≤6, 7, or 8–10). Models were adjusted for PSA values (log-transformed due to nonnormal distribution), race, age, and BMI. All analyses were performed using Stata v.11 (StataCorp, College Station, TX, USA), and an alpha level of <0.05 was considered as statistically significant.

## 3. Results

The clinical and demographic data are summarized in Table 1. The median patient age was 62.5 years (IQR 57–73 years). The median CCI was 3 (IQR 2–4), fifty-four patients (69%) were AA, and 44 patients (56%) were married. For 53 patients (68%) the PSA prior to biopsy was their first measurement, and the median PSA was 34.3 ng/mL (IQR 24.2–55.4 ng/mL). Sixty-six patients (85%) had a positive biopsy and the majority were Gleason 8–10 (*n* = 44, 67%).

**Figure 1:**
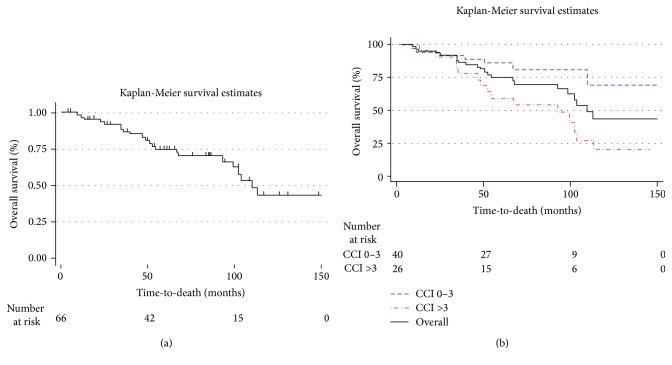
Kaplan-Meier survival estimates of men with positive prostate biopsy (*n* = 66) with regards to (a) overall survival and (b) Charlson Comorbidity Index (CCI).

Among the 66 patients with a positive biopsy, the most common treatment modality was androgen deprivation therapy (ADT)/bilateral simple orchiectomy (*n* = 37, 56%), followed by EBRT/ADT (*n* = 18, 27%), RP (*n* = 5, 8%), EBRT alone (*n* = 4, 6%), and no treatment (*n* = 2, 3%). The 5-year OS in men with positive prostate biopsy was 75% (95% CI 62, 85%) (Figure 1(a)); for patients with a CCI score of 0–3 and >3, 5-year OS was 86% (95% CI 69, 94%) and 59% (95% CI 36, 77%), respectively (*P* = 0.001) (Figure 1(b)). The median follow-up time of the cohort was 71.7 months (IQR 51.2–108.2). During followup there were 17 deaths (22%), of which six (35%) were PCa-specific deaths. Of the 27 patients who received definitive treatment, eight (30%) had BCR at a median of 14.4 months after treatment (median followup in this group-64.4 mos). Among these patients with BCR, four patients died (50%), of which one patient died of PCa.

On multivariate analysis, CCI (HR 1.52, 95% CI 1.19, 1.94, *P* = 0.001), PSA (HR 1.62, 95% CI 1.09, 2.42, *P* = 0.02), and Gleason score (HR 2.04, 95% CI 1.17, 3.56, *P* = 0.01) were independent predictors of OS among patients with positive biopsy (Table 2). Age (*P* = 0.15), race (*P* = 0.53), and marital status (*P* = 0.28) were not predictors of OS. When the association between CCI and positive biopsy was tested, CCI was not predictive of a positive biopsy (*P* = 0.50). However, on multivariable analysis CCI was associated with highergrade disease (Gleason 7 PCa—OR 4.06, 95% CI 1.04, 15.89; Gleason 8–10 PCa—OR 4.52, 95% CI 1.16, 17.54) (Table 3).

## 4. Discussion

Our study of men with PSA levels >20 ng/mL at time of biopsy shows that the patient's overall health status (based on CCI) was a significant independent predictor of higher-grade disease (Gleason score ≥ 7) and OS. However, other demographic characteristics such as race or marital status among these high-risk men were unrelated to OS. Although the sample cohort was small, we confirmed that Gleason score and PSA were associated with OS, verifying the cohort for detecting other factors that may be associated with OS such as CCI and marital status.

Comorbidity is an important factor to consider when counseling patients for cancer screening and treatment, as comorbidity may be associated with increased cancer risk and aggressive disease [20–23]. Furthermore, comorbidity in cancer patients may be a competing factor for overall survival [24–28]. In a recent study, Briganti et al. analyzed 3828 men treated with RP for high-risk PCa, assessing 10-year cancer specific and overall cause mortality stratified by CCI [27]. They found that age and CCI were major determinants of overall cause mortality, while the impact of CCI on cancer specific mortality was minimal. Rider et al. used the National Prostate Cancer Register of Sweden to identify 76,437 men treated with noncurative intent for PCa, analyzing 10-year mortality rates based on five disease risk groups (low, intermediate, high, regional metastases, and distant metastases) and stratified by CCI [28]. They found that comorbidity was a strong predictor of death, particularly in men <65 years of age. Subhazard ratios for non-PCa death comparing a CCI score ≥2 to a CCI score 0 were 6.1 in the low-risk, 9.3 in the intermediate-risk, and 5.1 in the high-risk group. Using the same CCI comparison, men 65–75 years of age had a threefold greater and men >75 years of age had a twofold greater risk of non-PCa death for higher CCI regardless of PCa risk stratification.

In the current study, CCI was an independent significant variable predicting OS, even in light of these patients being more likely to have Gleason score 7–10 disease. However, CCI may not be an updated, appropriate index for assessing patient comorbidity [29]. For example, the original, and still commonly used, CCI scoring index [5] gives autoimmune deficiency syndrome (AIDS) a greater weight (six points) than it probably should currently receive, given the improvements in AIDS treatment. Furthermore, CCI was developed only in hospitalized men [5], which may not be valid for most men receiving urologic treatment who are seen in the outpatient setting. Despite these potential limitations, CCI was still significant in our study cohort. Taken together, the above findings and the current study emphasize the need to risk stratify patients prior to screening, biopsy, and treatment of PCa.

Race and marital status did not predict OS among men undergoing prostate biopsy, perhaps secondary to the small sample size. Furthermore, we did not analyze the qualitative aspect of marriage, which may also influence whether marital status can modify outcomes among PCa patients. Alternatively, the negative association between marital status and OS may be secondary to the aggressive, high-risk PCa that may override any benefit from a stable marriage. The impact of marital status on clinicopathologic outcomes has been previously addressed in patients undergoing radical cystectomy for bladder cancer and patients undergoing radical prostatectomy for prostate cancer, identifying improved CSS and OS in married patients [15–18]. Abdollah et al. analyzed SEER for patients undergoing RP and identified 163, 697 patients with organ confined PCa [18]. They found that men who were SDW had more advanced stage at RP and higher causes specific and all-cause mortality compared to married men. Ultimately, patients without a support system associated with marriage may be at risk for poor outcomes and may require additional effort in order to maximize excellent clinical outcomes.

Limitations of the study include the inherent bias associated with retrospective collection and evaluation of data. Second, the sample size of this cohort is small and may be underpowered to detect differences in results that were negative. Thus, we cannot conclusively rule out the importance of marital status and race for men with PSA >20 ng/mL. Including all patients referred for biopsy regardless of PSA level may ultimately prove marital status to be a significant variable in clinical outcomes. A small sample size may also bias positive associations found in logistic regression studies, falsely increasing the magnitude of associations [30]. Finally, this is a single-center study; thus the way patients were clinically managed throughout their PCa treatment at our particular medical center may have contributed to the findings related to CCI. Strengths of this study include the equal access nature of the VA medical system, thus making the results more generalizable and analyzing men exposed to distinct treatments, thus avoiding the common selection bias of cohort studies of a particular treatment.

## 5. Conclusions

Patient comorbidity was an independent predictor of worse OS, highlighting the importance of a holistic approach to PCa treatment and integrating disease-specific PCa data with patient clinical status when counseling and deciding on treatment for high-risk patients. In this study, race and marital status were unrelated to OS among men with PSA >20 ng/mL at diagnosis. The impact of marital status and race on PCa diagnosis and outcomes still remains to be ascertained in large, prospective, and multicenter cohorts.

## Figures and Tables

**Table 1:** Clinical and demographic data (*n* = 78). Values are reported as median (interquartile range) or number (%).

Age (years)	62.5 (57–73)
Race	
AA	54 (69.2%)
Caucasian	24 (30.8%)
Marital Status	
Married	44 (56.4%)
SDW	33 (43.6%)
BMI (kg/m^2^)	27.5 (23.5–33.3)
CCI	3 (2–4)
PSA (ng/mL)	34.3 (24.2–55.4)
Prior PSA, yes	53 (67.9%)
Prior biopsy, yes	16 (20.5%)
Biopsy results, positive∗	66 (84.6%)
Gleason score (*n* = 66)	
6	5 (7.6%)
7	17 (25.8%)
8–10	44 (66.7%)
Clinical stage (*n* = 66)	
T1c	33 (50%)
T2a	8 (12.1%)
T2b	11 (16.7%)
T2c	7 (10.6%)
T3a	5 (7.6%)
T3b	2 (3%)

AA: African-American, BMI: body mass index, CCI: Charlson Comorbidity Index, PSA: prostate-specific antigen, SDW: single, divorced, or widowed.

∗One patient had two prior negative biopsies; one patient had one prior negative biopsy.

**Table 2:** Predictors of OS among men with positive biopsy^#^.

	HR (95% CI)	*P*-value
CCI	1.52 (1.19, 1.94)	0.001
African American versus Caucasian	1.42 (0.47, 4.36)	0.53
SDW versus married	0.59 (0.23, 1.54)	0.28
Gleason score∗	2.04 (1.17, 3.56)	0.01
Log-transformed PSA	1.62 (1.09, 2.42)	0.02
Age (years)	0.96 (0.91, 1.01)	0.15

HR: hazard ratio, SDW: single/divorced/widowed, PSA: prostate-specific antigen, CCI: Charlson Comorbidity Index.

^#^Cox proportional hazards model—adjusted for PSA (log-transformed), race, age, and BMI.

∗Introduced as a discrete variable in the model.

**Table 3:** Association of CCI with positive biopsy and tumor grade.

	Univariate (95% CI)	*P* value	Multivariate (95% CI)	*P* value
Positive biopsy∗ (OR)	1.13 (0.80, 1.59)	0.50	1.42 (0.75, 2.71)	0.28
Gleason score^†^ (OR)				
Gleason 6	1		1	
Gleason 7	2.37 (0.88, 6.37)	0.09	4.06 (1.04, 15.89)	0.04
Gleason 8–10	2.58 (0.97, 6.83)	0.06	4.52 (1.16, 17.54)	0.03

OR: odds ratio, CI: confidence interval, CCI: Charlson Comorbidity Index, PSA: prostate-specific antigen.

∗Logistic regression analysis; multivariable analysis adjusted for age, race, log-transformed PSA, and BMI.

^†^Multinomial logistic regression analysis; multivariable analysis adjusted for BMI, race, and log-transformed PSA.
